# Measurement Invariance of the Drivers of COVID-19 Vaccination Acceptance Scale: Comparison between Taiwanese and Mainland Chinese-Speaking Populations

**DOI:** 10.3390/vaccines9030297

**Published:** 2021-03-22

**Authors:** Ya-Chin Yeh, I-Hua Chen, Daniel K. Ahorsu, Nai-Ying Ko, Kuan-Lin Chen, Ping-Chia Li, Cheng-Fang Yen, Chung-Ying Lin, Mark D. Griffiths, Amir H. Pakpour

**Affiliations:** 1 Institute of Allied Health Sciences, College of Medicine, National Cheng Kung University, Tainan 701401, Taiwan; yehyachin@ms.szmc.edu.tw (Y.-C.Y.); klchen@mail.ncku.edu.tw (K.-L.C.); 2 Department of Occupational Therapy, Shu-Zen Junior College of Medicine and Management, Kaohsiung 82144, Taiwan; 3 School of Education Science, Minnan Normal University, Zhangzhou 363000, China; ahole.chen@gmail.com; 4 Department of Rehabilitation Sciences, Faculty of Health & Social Sciences, The Hong Kong Polytechnic University, Hung Hom, Hong Kong; daniel.ahorsu@connect.polyu.hk; 5 Department of Nursing, National Cheng Kung University Hospital, College of Medicine, National Cheng Kung University, Tainan 701401, Taiwan; nyko@mail.ncku.edu.tw; 6 International Doctoral Program in Nursing, Department of Nursing, College of Medicine, National Cheng Kung University, Tainan 701401, Taiwan; 7 Department of Occupational Therapy, College of Medicine, National Cheng Kung University, Tainan 701401, Taiwan; 8 Department of Physical Medicine and Rehabilitation, National Cheng Kung University Hospital, College of Medicine, National Cheng Kung University, Tainan 701401, Taiwan; 9 Department of Occupational Therapy, I-Shou University, Kaohsiung 824005, Taiwan; pingchia@isu.edu.tw; 10 Department of Psychiatry, Kaohsiung Medical University Hospital & School of Medicine College of Medicine, Kaohsiung Medical University, Kaohsiung 807378, Taiwan; 11 Department of Public Health, National Cheng Kung University Hospital, College of Medicine, National Cheng Kung University, Tainan 701401, Taiwan; 12 International Gaming Research Unit, Psychology Department, Nottingham Trent University, Nottingham NG14FQ, UK; mark.griffiths@ntu.ac.uk; 13 Department of Nursing, School of Health and Welfare, Jönköping University, 55318 Jönköping, Sweden; pakpour_amir@yahoo.com

**Keywords:** confirmatory factor analysis, COVID-19, measurement invariance, vaccine, university students, Drivers of COVID-19 Vaccination Acceptance Scale

## Abstract

The impacts of novel coronavirus disease-2019 (COVID-19) on human life continue to be serious. To control the spread of COVID-19, the production of effective vaccines is likely to be one of the best solutions. However, vaccination hesitancy may decrease individuals’ willingness to get vaccinated. The Drivers of COVID-19 Vaccination Acceptance Scale (DrVac-COVID19S) was recently developed to help healthcare professionals and researchers better understand vaccination acceptance. The present study examined whether DrVac-COVID19S is measurement invariant across different subgroups (Taiwanese vs. mainland Chinese university students; males vs. females; and health-related program majors vs. non-health-related program majors). Taiwanese (*n* = 761; mean age = 25.51 years; standard deviation (SD) = 6.42; 63.5% females) and mainland Chinese university students (*n* = 3145; mean age = 20.72 years; SD = 2.06; 50.2% females) were recruited using an online survey between 5 January and 21 February 2021. Factor structure and measurement invariance of the two DrVac-COVID19S scales (nine-item and 12-item) were tested using confirmatory factor analysis (CFA). The findings indicated that the DrVac-COVID19S had a four-factor structure and was measurement invariant across the subgroups. The DrVac-COVID19S’s four-factor structure was supported by the CFA results is a practical and valid instrument to quickly capture university students’ willingness to get COVID-19 vaccination. Moreover, the DrVac-COVID19S can be used to compare university students’ underlying reasons to get COVID-19 vaccination among different subgroups.

## 1. Introduction

Coronavirus disease 2019 (COVID-19) was identified as a worldwide pandemic in early 2020. Consequently, individuals globally have had their everyday life activities substantially impacted for over a year at the time of writing [1,2,3,4]. The impacts of COVID-19 have been great given that individuals’ physical, psychological, and social health have been jeopardized. This has been due to COVID-19′s impact both directly (e.g., the physical discomfort or physical impairment due to the COVID-19 infection) and/or indirectly (e.g., the policy of preventing COVID-19 infection such as lockdown that may impair an individual’s physical or psychosocial health) [5,6,7,8,9,10,11,12,13,14,15,16,17,18,19]. Unfortunately, no country is immune to the impacts of COVID-19 because many countries are still suffering from continued waves of COVID-19 outbreaks [4,7,20]. Expectations for controlling COVID-19 transmissions rely heavily on the vaccines that have been produced. Through countrywide vaccination programs, governments worldwide are likely to be able to control the COVID-19 outbreaks and significantly curtail the negative impact of COVID-19 and resume in-person global interactions.

Although governments and healthcare providers are pleased that effective COVID-19 vaccines have now been developed, an unresolved problem is the willingness of individuals to have them [21,22]. More specifically, the uptake rate of COVID-19 vaccination will influence whether global communities can be fully protected from COVID-19 and avoid future outbreaks [23,24,25]. When a significant number of individuals get vaccinated, the infection rate of COVID-19 can be controlled and will avoid further community outbreaks. To date, more than 160 candidate vaccines have been tested to respond to the urgent need for a COVID-19 vaccine [26,27,28]. Given that some of these have now shown to be effective in clinical trials, the next step in effective COVID-19 vaccination is to maximize the uptake rate of the effective vaccines. Therefore, information concerning individuals’ attitudes and considerations to get vaccinated should be investigated to promote individuals and communities to uptake COVID-19 vaccination.

The Drivers of COVID-19 Vaccination Acceptance Scale (DrVac-COVID19S) appears to be a good candidate instrument to assess individuals’ attitudes and considerations in getting vaccinated against COVID-19. The DrVac-COVID19S was adapted from a well-established instrument assessing influenza vaccination acceptance (i.e., Motors of Influenza Vaccination Acceptance Scale; MoVac-Flu Scale) [29]. Therefore, the DrVac-COVID19S shares the same cognitive model of empowerment (CME) as the MoVac-Flu Scale in understanding different traits described in the CME [29,30]. The CME proposes that empowerment is an intrinsic motivation that is determined by four cognitive traits for individuals to perform purposeful behaviors, such as vaccination uptake. These four cognitive traits are values (i.e., how an individual cares about the purpose of COVID-19 vaccination uptake), impacts (i.e., how an individual believes in the effects of COVID-19 vaccination uptake in preventing COVID-19 infection), knowledge (i.e., how an individual has knowledge regarding the COVID-19 vaccination uptake), and autonomy (i.e., how much an individual is confident and has control in getting COVID-19 vaccination if the individual wants to). In other words, when the CME is applied to understand an individual’s attitudes and considerations in getting vaccinated against COVID-19, the individual’s *values, perceived impacts, knowledge, and autonomy* are key traits for them to get vaccinated against COVID-19.

The factor structure of the DrVac-COVID19S has been found to fit well with the CME model among a mainland Chinese sample using the confirmatory factor analysis (CFA) [31]. In addition, two versions of the DrVac-COVID19S have been proposed: a nine-item DrVac-COVID19S with all items positively worded and a 12-item DrVac-COVID19S with nine items positively worded and three items negatively worded. Both versions fit well with the four-trait-factor structure in the CME model. Moreover, the 12-item DrVac-COVID19S fitted well with the CME model when the wording effects were controlled for in the CFA models [31]. However, aside from the aforementioned study, to the best of the present authors’ knowledge, no other psychometric evidence for the DrVac-COVID19S has been examined. Therefore, there is a need to further understand the psychometric properties of the DrVac-COVID19S.

In order to provide additional psychometric evidence of the DrVac-COVID19S, the present study examined the measurement invariance properties of the DrVac-COVID19S across subsamples. Measurement invariance is an important property that identifies whether the score difference of an instrument (e.g., the DrVac-COVID19S in the present study) detects the true differences between subsamples or whether the differences are due to various interpretations of the item descriptions in the instrument [32,33]. However, given that the DrVac-COVID19S was developed recently, no information regarding the measurement invariance of the DrVac-COVID19S has been reported. With knowledge of the measurement invariance of the DrVac-COVID19S, researchers, healthcare providers, and policymakers will have information to compare and evaluate the true differences in the acceptance of COVID-19 vaccination between different subsamples. In this regard, healthcare providers, researchers, and policymakers may use different findings between subsamples (e.g., males vs. females) to assist them in taking the best approach to promote uptake of COVID-19 vaccination [34]. When information concerning measurement invariance is not reported, it is not known whether the differences between subsamples are real differences or interpretation differences [35]. Therefore, from the perspective of psychometric testing, testing the measurement invariance of the DrVac-COVID19S across different subsamples is both important and necessary.

Consequently, the aims of the present study were to (i) reconfirm the factor structure of the DrVac-COVID19S and (ii) examine the measurement invariance of the DrVac-COVID19S across university students in three comparisons (i.e., Taiwan university students vs. mainland Chinese university students; male university students vs. female university students; university students majoring in a health-related program vs. university students not majoring in a health-related program). The present study focused on university students because they will be one of the last cohorts to get vaccinated because they are not generally clinically vulnerable. Therefore, they may not be fully aware of the importance of vaccination acceptance and/or will think they do not need to have a vaccine because they are such a low-risk group. In this regard, it is important to test the DrVac-COVID19S on this population to more deeply understand their attitudes and knowledge concerning COVID-19 vaccination.

## 2. Methods

### 2.1. Participants and Procedure

Institutional review boards (IRBs) of the Kaohsiung Medical University Chung-Ho Memorial Hospital (IRB ref: KMUHIRB-EXEMPT(I)-20200119) and the Jianxi Psychological Consultant Association (IRB ref: JXSXL-2020-DE22) approved the study protocol before data collection commenced. The study adopted a cross-sectional design, and all data collection used snowball sampling (a type of convenience sampling). For collecting data from Taiwanese university students, Google Forms was used to host an online survey, and the data were collected between 5 January and 1 February 2021. For collecting data from mainland Chinese university students, Sojump was used to host an online survey, and the data were collected between 5 January and 16 January 2021. More specifically, the link and Quick Response (QR) code of the *Google Forms* survey was sent out to the Taiwanese university students with assistance from university faculty members; the Sojump survey was sent out to mainland Chinese university students with assistance from university counselors. A total of 761 Taiwanese university students and 3145 mainland Chinese students participated in the present study.

The inclusion criteria for the present study’s participants were (i) currently enrolled at university and studying an undergraduate or postgraduate program and (ii) being aged 20 years or above (for Taiwanese students) or 18 years or above (for mainland Chinese students). The difference in ages was due to the Taiwan IRB requesting that Taiwanese students aged under 20 years needed to have parental consent to participate. The study information was clearly and thoroughly described at the beginning of the online survey, and only those who agreed to provide informed consent were able to participate in the survey. Moreover, the online surveys could not be submitted if any questions were left blank. Therefore, the present study had no missing data in either the Taiwanese and mainland Chinese samples.

### 2.2. The DrVac-COVID19S and Demographic Questionnaire

The DrVac-COVID19S (Appendix A) was adapted from the MoVac-Flu Scale with permission from Professor Vallée-Tourangeau, the developer of the MoVac-Flu Scale. The major difference between the DrVac-COVID19S and the MoVac-Flu Scale is that the MoVac-Flu Scale uses the word flu, and the DrVac-COVID19S uses the word COVID-19. The DrVac-COVID19S contains 12 items, where nine items are positively worded (items 1 to 6, 8, 9, and 12) and three items are negatively worded (items 7, 10, and 11). Therefore, the DrVac-COVID19S shares the same model of CME as the MoVac-Flu Scale in assessing an individual’s values, impacts, knowledge, and autonomy traits. The four traits can help healthcare providers and researchers to understand how an individual (i) cares about the purpose of COVID-19 vaccination uptake (*values*); (ii) believes in the effects of COVID-19 vaccination uptake in preventing COVID-19 infection (*impacts*); (iii) has knowledge regarding the COVID-19 vaccination uptake (*knowledge*); and (iv) is confident and has control in getting COVID-19 vaccination if the individual wants to (*autonomy*). Moreover, the 12 items comprise four traits corresponding to the CME model: items 3 (“It is important that I get the COVID-19 jab”), 6 (“The COVID-19 jab plays an important role in protecting my life and that of others”), and 8 (“The contribution of the COVID-19 jab to my health and well-being is very important”) comprise values; items 1 (“Vaccination is a very effective way to protect me against the COVID-19”), 4 (“Vaccination greatly reduces my risk of catching COVID-19), and 12 (“Getting the COVID-19 jab has a positive influence on my health”) comprise impacts; items 2 (“I know very well how vaccination protects me from the COVID-19”), 5 (“I understand how the flu jab helps my body fight the COVID-19 virus”), and 10 (“How the COVID-19 jab works to protect my health is a mystery to me”) comprise knowledge; and items 7 (“I feel under pressure to get the COVID-19 jab”), 9 (“I can choose whether to get a COVID-19 jab or not”), and 11 (“I get the COVID-19 jab only because I am required to do so”) comprise autonomy. All the items are rated using a seven-point Likert scale. After reverse coding, the negatively worded items (i.e., scoring for these items are from 1 (strongly agree) to 7 (strongly disagree)), a higher score in the DrVac-COVID19S indicates a higher level of COVID-19 vaccine acceptance.

The demographic questionnaire asked the participants’ sex (male or female), age (in years), educational level (undergraduate or postgraduate), and their major subject of study.

### 2.3. Data Analysis

**Figure 1:**
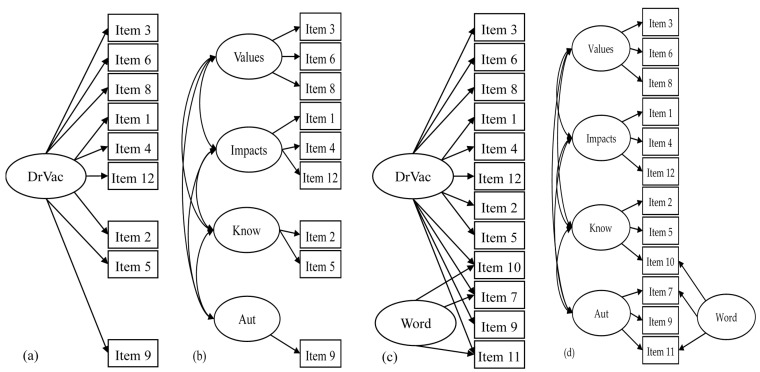
The four confirmatory factor analysis models for the Drivers of COVID-19 Vaccination Acceptance Scale (DrVac-COVID19S) tested in the present study. (**a**) One-trait-factor model using 9 positively worded items; (**b**) Four-trait-factor model using 9 positively worded items; (**c**) One-trait-factor and two-minus-one-method-factor model using all 12 items; (**d**) Four-trait-factor and two-minus-one-method-factor model using all 12 items. Note: error terms are not presented in the figure. DrVac = DrVac-COVID19S; Know = knowledge; Aut = autonomy; Word = negative wording effect.

Descriptive statistics, including means and frequencies, were firstly used to examine the participants’ characteristics and the participants’ scores on the DrVac-COVID19S. Moreover, χ^2^ test and independent *t*-test were used to examine differences in participant characteristics and the DrVac-COVID19S item score differences between Taiwanese and mainland Chinese university students. The present study tested four-factor structures for the DrVac-COVID19S, including the nine-item one-trait-factor structure (using nine positively worded items), nine-item four-trait-factor structure (using nine positively worded items), 12-item one-trait-factor with two-minus-one-method-factor structure (method factor constructed on negatively worded items), and 12-item four-trait-factor with two-minus-one-method factor structure (method factor constructed on negatively worded items) (see Figure 1).

The four-factor structures were evaluated using CFA. Moreover, the four trait factors are the four traits described in the CME model (i.e., value, impact, knowledge, and autonomy). The two-minus-one-method-factor structure was tested because the four-trait-factor with a two-method-factor structure is more complicated and usually has problems in convergence due to the violation of the parsimony principle for a CFA model [36].

All the CFA models were examined using the commonly used fit indices of comparative fit index (CFI), Tucker–Lewis index (TLI), root mean square error of approximation (RMSEA), and standardized root mean squared residual (SRMR). A supported model should have CFI and TLI > 0.95 with RMSEA and SRMR < 0.08 [37,38]. More specifically, the CFI and TLI indicate to what extent the proposed model is better than the worst model (i.e., the null model in the CFA). Therefore, high values for CFI and TLI are expected. The RMSEA indicates whether the model fulfills the parsimony in the psychometric testing and a smaller value indicates more parsimony of the proposed model. The SRMR indicates the residuals in the proposed model and, therefore, a small value is expected. Although a non-significant χ^2^ also indicates a supported model, the χ^2^ test was not applied due to its sensitivity to large sample sizes [39]. Aside from the CFI, TLI, RMSEA, and SRMR, the four models were compared using χ^2^ difference tests to understand which factor structure significantly outperformed the others [40]. More specifically, when the χ^2^ difference test is significant, the model with a lower value of χ^2^ is significantly better. All the CFAs were analyzed using the diagonally weighted least square estimator.

Measurement invariance of the four-factor structures was separately examined for the following subsamples: Taiwanese vs. mainland Chinese; males vs. females; and health-related major students vs. non-health-related major students. With the test of measurement invariance, it can be confirmed whether the factor structure of the DrVac-COVID19 was interpreted in the same way across these subsamples. This further strengthens the evidence regarding the DrVac-COVID19S’s factor structure. Three tests of measurement invariance were performed. For each test of measurement invariance, three nested models were constructed: (i) a configural model, which freely estimated all the factor loadings and item intercepts across the two subsamples (e.g., Taiwanese and mainland Chinese); (ii) a factor-loading-constrained model, which constrained the factor loadings equally between the two subsamples and freely estimated the item intercepts across the two subsamples; and (iii) a factor-loading and item-intercept-constrained model, which constrained the factor loadings and item intercepts equally between the two subsamples [38,41,42]. The aforementioned CFI, TLI, RMSEA, and SRMR were used to examine whether a configural model was supported. Moreover, ΔCFI, ΔRMSEA, and ΔSRMR were used to examine whether the more constrained model was equivalent to the less constrained model. That is, the configural model (a less constrained model) was compared with the factor-loading-constrained model (a more constrained model), and the factor-loading-constrained model (a less constrained model) was compared with the factor-loading and item-intercept-constrained model (a more constrained model). ΔCFI > −0.01, ΔRMSEA < 0.015, and ΔSRMR < 0.03 (for factor loading) or < 0.01 (for item intercept) indicate that the two nested models are equivalent and therefore the measurement invariance across the two subsamples is supported for the tested factor model [43]. More specifically, when the values fulfill the aforementioned cutoffs, this indicates that the two compared models are equally good. IBM SPSS 24.0 (IBM Corp., Armonk, NY, USA) and LISREL (Scientific Software International, Lincolnwood, IL, USA) were used for data analysis.

## 3. Results

The participants’ characteristics are presented in Table 1. These showed that 761 Taiwanese university students (483 (63.5%) females; mean (SD) age = 25.51 years (6.42) years) and 3145 mainland Chinese university students (1493 (50.2%) females; mean (SD) age = 20.72 years (2.06)) participated in the present study. Most of the participants were studying in an undergraduate program (458 (60.2%) Taiwanese and 3026 (96.2%) mainland Chinese) and studying in a non-health-related program (286 (37.6%) Taiwanese and 241 (7.7%) mainland Chinese). All these characteristics were significantly different between Taiwanese and mainland Chinese students. Moreover, all the DrVac-COVID19S item scores, except for item 7, were significantly different between Taiwanese and mainland Chinese students: Taiwanese students had significantly higher scores in items 9 to 11, and mainland Chinese students had significantly higher scores in items 1 to 6, 8, and 12.

The CFA results showed that the four proposed factor structures were relatively good across all the subsamples (including Taiwanese vs. mainland Chinese, males vs. females, and students majoring in health-related programs vs. those majoring in non-health-related programs), except for slightly high RMSEAs (0.099 in nine-item one-trait-factor structure for Taiwanese and 0.090 in students majoring in health-related programs; 0.107 in 12-item one-trait-factor and two-minus-one-method- factor structure for Taiwanese and 0.095 in students majoring in health-related programs). Moreover, the χ^2^ difference tests demonstrated that the nine-item four-trait-factor model significantly fitted better than the nine-item one-trait-factor model (*p* < 0.001); the 12-item four-trait-factor and two-minus-one-method-factor model significantly fitted better than the 12-item one-trait-factor and two-minus-one-method-factor model (*p* < 0.001). Therefore, four-trait-factor models appeared to outperform one-trait-factor models irrespective of item numbers for the DrVac-COVID19S (Table 2).

Measurement invariance was subsequently carried to understand which factor structure(s) was (were) invariant across subsamples (Table 3). All the configural models had excellent fit indices (CFI = 0.984 to 0.998; RMSEA = 0.043 to 0.076; SRMR = 0.018 to 0.047) for all the factor structure models in all the subsamples. Most of the fit indices in measurement invariance testing supported that the four-factor structures (including nine-item one-trait-factor model, nine-item four-trait-factor model, 12-item one-trait-factor and two-minus-one-method-factor model, and 12-item four-trait-factor and two-minus-one-method-factor model) were invariant across regions (Taiwan vs. mainland China), sex (male vs. female), and study majors (health-related vs. non-health-related).

## 4. Discussion

The present study replicated the findings of the DrVac-COVID19S factor structure in a previous study [31]. More specifically, the DrVac-COVIDS19S corresponded well with the four-trait-factor portrayed by the CME [29,30] (i.e., that values, impacts, knowledge, and autonomy are important traits in explaining an individual’s underlying drivers to accept COVID-19 vaccination). Similar to prior findings on the factor structure of the DrVac-COVID19S [31], the present study found that the 12-item version of DrVac-COVID19S should be controlled for its wording effects when evaluating its factor structure. Moreover, the present study found that the four-trait-factor for the nine-item DrVac-COVID19S and the four-trait-factor and two-minus-one-method factor for the 12-item DrVac-COVID19S were supported consistently across different subsamples in the present study. Therefore, the trait-factor structure of the DrVac-COVID19S (either nine-item or 12-item version) can tentatively be concluded to be a four-trait-factor structure corresponding to the CME.

Although the present study’s findings agreed with the prior findings on the DrVac-COVID19S factor structure [31], prior psychometric evidence concerning the MoVac-Flu Scale (i.e., the instrument that was used and adapted to develop the DrVac-COVID19S) supports a single trait-factor [29]. The different findings in the factor structures are likely due to the different statistical methods used between the studies. More specifically, the factor structure of the MoVac-Flu Scale was tested using exploratory factor analysis, and the DrVac-COVID19S was tested using CFA [31]. Exploratory factor analysis cannot test the factor structure via a hypothesized model, and it is subjective and arbitrary to decide the number of factors in the structure. In contrast, CFA examines whether a factor structure fits well with a hypothesized model (e.g., the present study proposed a four-trait-factor structure). Therefore, different findings may be obtained between exploratory factor analysis and CFA. Nevertheless, the CFA findings in the present study and the previous DrVac-COVID19S study [31] demonstrated acceptable fit indices for the one-trait-factor structure of the DrVac-COVID19S. This implies that the DrVac-COVID19S fits well in two types of trait-factor structures (i.e., one trait-factor and four trait-factors) [30]. More specifically, the single trait in the one-trait-factor structure is the empowerment trait that is constructed by values, impacts, knowledge, and autonomy. The traits in the four-trait-factor structure are values, impacts, knowledge, and autonomy.

The four-trait-factor structure of the DrVac-COVID19S was further found to be equivalent (or invariant) across three different subsamples (i.e., Taiwanese vs. mainland Chinese, males vs. females, and health-related majors vs. non-health-related majors). These findings indicate that the DrVac-COVID19S has a consistent factor structure and is interpreted similarly across different subsamples. Given the robust findings, healthcare providers, researchers, and policymakers can use the DrVac-COVID19S to better understand the differences of willingness to uptake COVID-19 vaccination between subsamples. In other words, the present study’s results demonstrate there are meaningful comparisons between subsamples in the DrVac-COVID19S assessed willingness to uptake COVID-19 vaccination. Based on the supported measurement invariance, the aforementioned stakeholder groups can confidently use the DrVac-COVID19S to compare acceptance of COVID-19 vaccination uptake between the subsampled groups (i.e., Taiwanese vs. mainland Chinese, males vs. females, and health-related majors vs. non-health-related majors) and subsequently design more effective programs to promote COVID-19 vaccination uptake.

With the development of the DrVac-COVID19S, researchers, healthcare providers, and policymakers can further explore the underlying reasons regarding lower levels of motivation to get COVID-19 vaccination. More specifically, using the four traits mentioned in the CME, researchers, healthcare providers, and policymakers can examine whether an individual’s low willingness to get vaccinated against COVID-19 is due to *values, perceived impacts, knowledge, autonomy*, or any combination of the four traits. After obtaining such information, appropriate policies or treatment programs can be developed to improve the willingness to get vaccinated against COVID-19. For example, if an individual’s knowledge is the reason for their low willingness to get vaccinated, education programs concerning COVID-19 vaccination and methods to distribute such knowledge are potential ways to improve the willingness to get vaccinated. Although some evidence has supported the high willingness of COVID-19 vaccinating uptake across individuals in different countries (e.g., [28,44,45,46,47]), some studies still reported low acceptance [48,49,50,51]. Therefore, increasing the willingness of COVID-19 vaccination uptake among individuals is still necessary, and this can be facilitated by using the DrVac-COVID19S for assessment to better understand individuals’ attitudes. More specifically, the DrVac-COVID19S provides detailed information regarding the underlying mechanisms as to why an individual is willing or unwilling to get vaccinated against COVID-19. With such information, programs can be designed to increase individuals’ willingness to get vaccinated. For example, the MoVac-Flu Scale (i.e., the instrument on which the DrVac-COVID19S is based) has been used to explore and understand the reasons why European healthcare workers accept flu vaccination [51].

There are some limitations to the present study. First, the present study only recruited university students of a relatively young age (25.51 years among Taiwanese participants and 20.72 among mainland Chinese participants). Therefore, the psychometric findings from the present study cannot be generalized to a population other than young adults and not to children, adolescents, middle-aged, and older adults. Following this limitation, the response rate of the present study was unknown, given that individuals were invited to participate via snowball sampling. Therefore, the present study’s findings may be biased due to this recruitment method. Second, the present study’s participants were recruited from Taiwan and mainland China, and the generalizability of the present findings cannot be applied to other Chinese-speaking populations residing in other countries (e.g., those that live in Hong Kong as well as some populations in Malaysia and Singapore). Similarly, the present findings cannot be generalized to any populations in a Western country. Future studies on other ethnic populations or citizens residing in different countries are needed to increase the psychometric evidence concerning the DrVac-COVID19S. Third, other psychometric properties of the DrVac-COVID19S were not examined in the present study. For example, no external criteria were used to assess the concurrent validity, and no test-retest reliability was examined. Therefore, future studies testing other properties of the DrVac-COVID19S are needed for researchers, healthcare providers, and policymakers to better understand the features of the DrVac-COVID19S. Such research would provide better insights regarding how to use the DrVac-COVID19S.

## 5. Conclusions

The present study found that the DrVac-COVID19S is a practical and valid instrument to quickly capture university students’ willingness to get COVID-19 vaccination. Moreover, the DrVac-COVID19S corresponds to the four traits described in the CME, and that can help researchers, healthcare providers, and policymakers to have some insights into university students’ underlying reasons to get COVID-19 vaccination. Nevertheless, further psychometric evidence on the DrVac-COVID19S is needed to provide a comprehensive and thorough evaluation of this instrument and prompt its use in the critical period of promoting the uptake of COVID-19 vaccination.

## Figures and Tables

**Table 1:** Participants’ characteristics and item score in the Drivers of COVID-19 Vaccination Acceptance Scale (DrVac-COVID19S).

Variables	M (SD) or *n* (%)		*t* or χ^2^ *(p*-Value)
	Taiwan (*n* = 761)	Mainland China (*n* = 3145)	
Sex (female)	483 (63.5%)	1493 (50.2%)	43.45 (<0.01)
Age	25.51 (6.42)	20.72 (2.06)	19.58 (<0.01)
Education level (undergraduate)	458 (60.2%)	3026 (96.2%)	847.54 (<0.01)
Education (health-related)	286 (37.6%)	241 (7.7%)	469.94 (<0.01)
DrVac-COVID19S			
Item 1	5.08 (1.24)	5.76 (1.16)	14.29 (<0.01)
Item 2	4.86 (1.43)	5.62 (1.24)	13.35 (<0.01)
Item 3	5.19 (1.44)	5.93 (1.14)	13.21 (<0.01)
Item 4	5.18 (1.35)	5.94 (1.08)	14.38 (<0.01)
Item 5	4.91 (1.52)	5.62 (1.28)	11.87 (<0.01)
Item 6	5.40 (1.31)	6.00 (1.06)	11.75 (<0.01)
Item 7 ^a^	4.89 (1.39)	4.85 (1.61)	0.68 (0.50)
Item 8	5.23 (1.29)	5.88 (1.14)	12.76 (<0.01)
Item 9	5.92 (1.06)	5.78 (1.24)	3.27 (<0.01)
Item 10 ^a^	5.20 (1.40)	4.78 (1.65)	7.09 (<0.01)
Item 11 ^a^	4.62 (1.54)	4.43 (1.77)	2.95 (<0.01)
Item 12	5.09 (1.21)	5.42 (1.39)	6.58 (<0.01)

^a^ Reverse-coded items (scoring for these items are from 1 (strongly agree) to 7 (strongly disagree)). Item 1: Vaccination is a very effective way to protect me against COVID-19. Item 2: I know very well how vaccination protects me from COVID-19. Item 3: It is important that I get the COVID-19 jab. Item 4: Vaccination greatly reduces my risk of catching COVID-19. Item 5: I understand how the flu jab helps my body fight the COVID-19 virus. Item 6: The COVID-19 jab plays an important role in protecting my life and that of others. Item 7: I feel under pressure to get the COVID-19 jab. Item 8: The contribution of the COVID-19 jab to my health and well-being is very important. Item 9: I can choose whether to get a COVID-19 jab or not. Item 10: How the COVID-19 jab works to protect my health is a mystery to me. Item 11: I get the COVID-19 jab only because I am required to do so. Item 12: Getting the COVID-19 jab has a positive influence on my health.

**Table 2:** Confirmatory factor analysis results of the Drivers of COVID-19 Vaccination Acceptance Scale (DrVac-COVID19S) in different subsamples.

Subsample	Nine-Item DrVac-COVID19S		12-Item DrVac-COVID19S	
Fit Indices	One-Factor	Four-Factor	One-Factor ^a^	Four-Factor ^a^
**Taiwan**				
χ^2^ (*df*)/*p*-value	212.59 (25)/<0.001	42.91 (20)/0.002	472.70 (49)/<0.001	195.52 (43)/<0.001
CFI	**0.984**	**0.998**	**0.968**	**0.989**
TLI	**0.977**	**0.997**	**0.957**	**0.982**
RMSEA	0.099	**0.039**	0.107	**0.068**
SRMR	**0.051**	**0.016**	**0.063**	**0.048**
**Mainland China**				
χ^2^ (*df*)/*p*-value	269.73 (25)/<0.001	163.21 (20)/<0.001	702.93 (49)/<0.001	578.78 (43)/<0.001
CFI	**0.995**	**0.997**	**0.989**	**0.991**
TLI	**0.993**	**0.995**	**0.985**	**0.986**
RMSEA	**0.056**	**0.048**	**0.065**	**0.063**
SRMR	**0.026**	**0.021**	**0.043**	**0.040**
**Male**				
χ^2^ (*df*)/*p*-value	204.47 (25)/<0.001	116.46 (20)/<0.001	561.18. (49)/<0.001	459.02(43)/<0.001
CFI	**0.994**	**0.997**	**0.986**	**0.989**
TLI	**0.992**	**0.994**	**0.981**	**0.983**
RMSEA	**0.062**	**0.051**	**0.075**	**0.072**
SRMR	**0.029**	**0.022**	**0.047**	**0.044**
**Female**				
χ^2^ (*df*)/*p*-value	267.78 (25)/<0.001	83.64 (20)/<0.001	593.97 (49)/<0.001	339.13 (43)/<0.001
CFI	**0.992**	**0.998**	**0.984**	**0.991**
TLI	**0.989**	**0.996**	**0.979**	**0.987**
RMSEA	**0.069**	**0.039**	**0.073**	**0.058**
SRMR	**0.029**	**0.017**	**0.047**	**0.041**
**Health major**				
χ^2^ (*df*)/*p*-value	130.44 (25)/<0.001	23.49 (20)/0.27	279.40 (49)/<0.001	122.65 (43)/<0.001
CFI	**0.988**	**0.999**	**0.976**	**0.992**
TLI	**0.983**	**0.999**	**0.968**	**0.988**
RMSEA	**0.090**	**0.018**	**0.095**	**0.059**
SRMR	**0.036**	**0.016**	**0.055**	**0.046**
**Non-health major**				
χ^2^ (*df*)/*p*-value	331.28 (25)/<0.001	154.55 (20)/<0.001	882.10 (49)/<0.001	661.03 (43)/<0.001
CFI	**0.994**	**0.997**	**0.986**	**0.990**
TLI	**0.992**	**0.995**	**0.982**	**0.985**
RMSEA	**0.060**	**0.045**	**0.071**	**0.065**
SRMR	**0.028**	**0.020**	**0.047**	**0.043**

CFI = comparative fit index; TLI = Tucker–Lewis index; RMSEA = root mean square error of approximation; SRMR = standardized root mean squared residual. Excellent fit values are in **bold**; i.e., CFI and TLI > 0.95; RMSEA and SRMR < 0.08. ^a^ Using correlated trait correlated method minus one model to control wording effects.

**Table 3:** Measurement invariance testing across subsamples in the structure of the Drivers of COVID-19 Vaccination Acceptance Scale (DrVac-COVID19S).

Model (Subsamples)	Nine-Item DrVac-COVID19S		12-Item DrVac-COVID19S	
Fit Indices	One-Factor	Four-Factor	One-Factor ^a^	Four-Factor ^a^
**Configural (Taiwan vs. China)**				
χ^2^ (*df*)/*p*-value	482.57 (50)/<0.001	207.81(40)/<0.001	1171.60 (98)/<0.001	771.18 (86)/<0.001
CFI	**0.993**	**0.997**	**0.984**	**0.990**
RMSEA	**0.067**	**0.046**	**0.075**	**0.064**
SRMR	**0.026**	**0.021**	**0.042**	**0.039**
**Loading constrained (Taiwan vs. China)**				
Δχ^2^ (*df*)/*p*-value	153.75 (8)/<0.001	32.55 (5)/<0.001	207.93 (13)/<0.001	182.24 (15)/<0.001
ΔCFI	**−0.002**	**0.000**	**−0.003**	**−0.002**
ΔRMSEA	**0.004**	**0.001**	**0.002**	**0.002**
ΔSRMR	**0.008**	**0.003**	**0.006**	**0.004**
**Loadings and intercepts constrained (Taiwan vs. China)**				
Δχ^2^ (*df*)/*p*-value	198.23 (8)/<0.001	500.43 (5)/<0.001	252.63 (10)/<0.001	143.47 (7)/<0.001
ΔCFI	**−0.004**	−0.030	**−0.002**	**−0.002**
ΔRMSEA	**0.006**	0.037	**0.003**	**0.002**
ΔSRMR	**0.009**	**−0.008**	0.011	0.043
**Configural (male vs. female)**				
χ^2^ (*df*)/*p*-value	466.72(50)/<0.001	201.80 (40)/<0.001	1152.51(98)/<0.001	806.23 (86)/<0.001
CFI	**0.994**	**0.997**	**0.986**	**0.991**
RMSEA	**0.065**	**0.046**	**0.074**	**0.066**
SRMR	**0.029**	**0.018**	**0.047**	**0.041**
**Loading constrained (male vs. female)**				
Δχ^2^ (*df*)/*p*-value	55.59 (8)/<0.001	23.07 (5)/<0.001	117.06 (13)/<0.001	109.59 (15)/<0.001
ΔCFI	**−0.001**	**0.000**	**−0.001**	**−0.001**
ΔRMSEA	**−0.001**	**−0.001**	**−0.001**	**−0.002**
ΔSRMR	**0.007**	**0.003**	**0.026**	**0.027**
**Loadings and intercepts constrained (male vs. female)**				
Δχ^2^ (*df*)/*p*-value	236.36 (8)/<0.001	186.10 (5)/<0.001	143.41 (10)/<0.001	63.20 (7)/<0.001
ΔCFI	−0.018	**−0.010**	**−0.003**	**−0.002**
ΔRMSEA	**0.009**	0.016	**0.001**	**0.000**
ΔSRMR	**−0.023**	**−0.015**	**−0.010**	**−0.021**
**Configural (health vs. non-health)**				
χ^2^ (*df*)/*p*-value	476.22 (50)/<0.001	186.48 (40)/<0.001	1189.99 (98)/<0.001	803.93 (86)/<0.001
CFI	**0.994**	**0.998**	**0.985**	**0.990**
RMSEA	**0.066**	**0.043**	**0.076**	**0.065**
SRMR	**0.028**	**0.020**	**0.047**	**0.043**
**Loading constrained (health vs. non-health)**				
Δχ^2^ (*df*)/*p*-value	60.75 (8)/<0.001	21.24 (5)/<0.001	97.28 (13)/<0.001	116.93 (15)/<0.001
ΔCFI	**−0.001**	**0.000**	**−0.001**	**−0.001**
ΔRMSEA	**−0.001**	**0.000**	**−0.002**	**−0.001**
ΔSRMR	**0.001**	**0.001**	**0.001**	**0.001**
**Loadings and intercepts constrained (health vs. non-health)**				
Δχ^2^ (*df*)/*p*-value	105.16 (8)/<0.001	388.86 (5)/<0.001	165.84 (10)/<0.001	72.71 (7)/<0.001
ΔCFI	**−0.002**	**−0.026**	**−0.003**	**−0.001**
ΔRMSEA	**0.002**	0.032	**0.001**	**0.001**
ΔSRMR	**0.003**	**−0.013**	**0.005**	0.025

CFI = comparative fit index; TLI = Tucker–Lewis index; RMSEA = root mean square error of approximation; SRMR = standardized root mean square residual. Excellent fit values are in **bold**; i.e., CFI and TLI > 0.95; RMSEA and SRMR < 0.08. Supported measurement invariance values are in **bold**; i.e., ΔCFI > −0.01; ΔRMSEA < 0.015; ΔSRMR < 0.03 (for factor loading) or < 0.01 (for item intercept). ^a^ Using correlated trait correlated method minus one model to control wording effects.

## Data Availability

The data will be available upon reasonable request to the corresponding authors.

## Appendix A. Drivers of COVID-19 Vaccination Acceptance Scale

**English version (items underlined are reverse-coded items)**

1. Vaccination is a very effective way to protect me against COVID-19.

Strongly disagree (1)

Disagree (2)

Slightly disagree (3)

Neither disagree nor agree (4)

Slightly agree (5)

Agree (6)

Strongly agree (7)

2. I know very well how vaccination protects me from COVID-19.

Strongly disagree (1)

Disagree (2)

Slightly disagree (3)

Neither disagree nor agree (4)

Slightly agree (5)

Agree (6)

Strongly agree (7)

3. It is important that I get the COVID-19 jab.

Strongly disagree (1)

Disagree (2)

Slightly disagree (3)

Neither disagree nor agree (4)

Slightly agree (5)

Agree (6)

Strongly agree (7)

4. Vaccination greatly reduces my risk of catching COVID-19.

Strongly disagree (1)

Disagree (2)

Slightly disagree (3)

Neither disagree nor agree (4)

Slightly agree (5)

Agree (6)

Strongly agree (7)

5. I understand how the flu jab helps my body fight the COVID-19 virus.

Strongly disagree (1)

Disagree (2)

Slightly disagree (3)

Neither disagree nor agree (4)

Slightly agree (5)

Agree (6)

Strongly agree (7)

6. The COVID-19 jab plays an important role in protecting my life and that of others.

Strongly disagree (1)

Disagree (2)

Slightly disagree (3)

Neither disagree nor agree (4)

Slightly agree (5)

Agree (6)

Strongly agree (7)

7. I feel under pressure to get the COVID-19 jab.

Strongly disagree (7)

Disagree (6)

Slightly disagree (5)

Neither disagree nor agree (4)

Slightly agree (3)

Agree (2)

Strongly agree (1)

8. The contribution of the COVID-19 jab to my health and well-being is very important.

Strongly disagree (1)

Disagree (2)

Slightly disagree (3)

Neither disagree nor agree (4)

Slightly agree (5)

Agree (6)

Strongly agree (7)

9. I can choose whether to get a COVID-19 jab or not.

Strongly disagree (1)

Disagree (2)

Slightly disagree (3)

Neither disagree nor agree (4)

Slightly agree (5)

Agree (6)

Strongly agree (7)

10. How the COVID-19 jab works to protect my health is a mystery to me.

Strongly disagree (7)

Disagree (6)

Slightly disagree (5)

Neither disagree nor agree (4)

Slightly agree (3)

Agree (2)

Strongly agree (1)

11. I get the COVID-19 jab only because I am required to do so.

Strongly disagree (7)

Disagree (6)

Slightly disagree (5)

Neither disagree nor agree (4)

Slightly agree (3)

Agree (2)

Strongly agree (1)

12. Getting the COVID-19 jab has a positive influence on my health.

Strongly disagree (1)

Disagree (2)

Slightly disagree (3)

Neither disagree nor agree (4)

Slightly agree (5)

Agree (6)

Strongly agree (7)

**Simplified Chinese version (items underlined are reverse-coded items)**

1.「注射疫苗将能非常有效地保护我免于感染新冠肺炎。」

完全不同意 (1)

很不同意 (2)

有一点不同意 (3)

没同意也没不同意 (4)

有一点同意 (5)

很同意 (6)

完全同意 (7)

2.「我非常清楚疫苗将会如何保护我免于感染新冠肺炎。」

完全不同意 (1)

很不同意 (2)

有一点不同意 (3)

没同意也没不同意 (4)

有一点同意 (5)

很同意 (6)

完全同意 (7)

3.「对我来说，注射新冠肺炎疫苗将会是重要的事。」

完全不同意 (1)

很不同意 (2)

有一点不同意 (3)

没同意也没不同意 (4)

有一点同意 (5)

很同意 (6)

完全同意 (7)

4.「注射疫苗将能大幅降低我感染新冠肺炎的机会。」

完全不同意 (1)

很不同意 (2)

有一点不同意 (3)

没同意也没不同意 (4)

有一点同意 (5)

很同意 (6)

完全同意 (7)

5.「我了解疫苗将通过何种机制来帮助我的身体对抗新冠肺炎病毒。」

完全不同意 (1)

很不同意 (2)

有一点不同意 (3)

没同意也没不同意 (4)

有一点同意 (5)

很同意 (6)

完全同意 (7)

6.「新冠肺炎疫苗将会在保护我和其他人的生命上扮演重要角色。」

完全不同意 (1)

很不同意 (2)

有一点不同意 (3)

没同意也没不同意 (4)

有一点同意 (5)

很同意 (6)

完全同意 (7)

7.「我预测未来我将会在注射新冠肺炎疫苗这件事情上感受到压力。」

完全不同意 (7)

很不同意 (6)

有一点不同意 (5)

没同意也没不同意 (4)

有一点同意 (3)

很同意 (2)

完全同意 (1)

8.「新冠肺炎疫苗将会对于我的健康和幸福有着重要贡献。」

完全不同意 (1)

很不同意 (2)

有一点不同意 (3)

没同意也没不同意 (4)

有一点同意 (5)

很同意 (6)

完全同意 (7)

9.「未来我会自己决定是否要注射新冠肺炎疫苗。」

完全不同意 (1)

很不同意 (2)

有一点不同意 (3)

没同意也没不同意 (4)

有一点同意 (5)

很同意 (6)

完全同意 (7)

10.「新冠肺炎疫苗将会如何保护我的健康，对我来说还是谜。」

完全不同意 (7)

很不同意 (6)

有一点不同意 (5)

没同意也没不同意 (4)

有一点同意 (3)

很同意 (2)

完全同意 (1)

11.「未来我将只有在被要求要注射时才会去注射新冠肺炎疫苗。」

完全不同意 (7)

很不同意 (6)

有一点不同意 (5)

没同意也没不同意 (4)

有一点同意 (3)

很同意 (2)

完全同意 (1)

12.「注射新冠肺炎疫苗将会对我的健康有正面的影响。」

完全不同意 (1)

很不同意 (2)

有一点不同意 (3)

没同意也没不同意 (4)

有一点同意 (5)

很同意 (6)

完全同意 (7)

**Traditional Chinese version (items underlined are reverse-coded items)**

1.「注射疫苗將能大幅降低我感染新冠肺炎的機會。」

完全不同意 (1)

很不同意 (2)

有一點不同意 (3)

沒同意也沒不同意 (4)

有一點同意 (5)

很同意 (6)

完全同意 (7)

2.「我非常清楚疫苗將會如何保護我免於感染新冠肺炎。」

完全不同意 (1)

很不同意 (2)

有一點不同意 (3)

沒同意也沒不同意 (4)

有一點同意 (5)

很同意 (6)

完全同意 (7)

3.「對我來說，注射新冠肺炎疫苗將會是重要的事。」

完全不同意 (1)

很不同意 (2)

有一點不同意 (3)

沒同意也沒不同意 (4)

有一點同意 (5)

很同意 (6)

完全同意 (7)

4.「注射疫苗將能大幅降低我感染新冠肺炎的機會。」

完全不同意 (1)

很不同意 (2)

有一點不同意 (3)

沒同意也沒不同意 (4)

有一點同意 (5)

很同意 (6)

完全同意 (7)

5.「我了解疫苗將經由何種機制來幫助我的身體對抗新冠肺炎病毒。」

完全不同意 (1)

很不同意 (2)

有一點不同意 (3)

沒同意也沒不同意 (4)

有一點同意 (5)

很同意 (6)

完全同意 (7)

6.「新冠肺炎疫苗將會在保護我和其他人的生命上扮演重要角色。」

完全不同意 (1)

很不同意 (2)

有一點不同意 (3)

沒同意也沒不同意 (4)

有一點同意 (5)

很同意 (6)

完全同意 (7)

7.「我預測未來我將會在注射新冠肺炎疫苗這件事情上感受到壓力。」

完全不同意 (7)

很不同意 (6)

有一點不同意 (5)

沒同意也沒不同意 (4)

有一點同意 (3)

很同意 (2)

完全同意 (1)

8.「新冠肺炎疫苗將會對於我的健康和幸福有著重要貢獻。」

完全不同意 (1)

很不同意 (2)

有一點不同意 (3)

沒同意也沒不同意 (4)

有一點同意 (5)

很同意 (6)

完全同意 (7)

9.「未來我將會幫自己決定是否要注射新冠肺炎疫苗。」

完全不同意 (1)

很不同意 (2)

有一點不同意 (3)

沒同意也沒不同意 (4)

有一點同意 (5)

很同意 (6)

完全同意 (7)

10.「新冠肺炎疫苗將會如何保護我的健康，對我來說還是謎。」

完全不同意 (7)

很不同意 (6)

有一點不同意 (5)

沒同意也沒不同意 (4)

有一點同意 (3)

很同意 (2)

完全同意 (1)

11.「未來我將只有在被要求要注射時才會去注射新冠肺炎疫苗。」

完全不同意 (7)

很不同意 (6)

有一點不同意 (5)

沒同意也沒不同意 (4)

有一點同意 (3)

很同意 (2)

完全同意 (1)

12.「注射新冠肺炎疫苗將會對我的健康有正面的影響。」

完全不同意 (1)

很不同意 (2)

有一點不同意 (3)

沒同意也沒不同意 (4)

有一點同意 (5)

很同意 (6)

完全同意 (7)
